# Theabrownin Induces Cell Apoptosis and Cell Cycle Arrest of Oligodendroglioma and Astrocytoma in Different Pathways

**DOI:** 10.3389/fphar.2021.664003

**Published:** 2021-04-30

**Authors:** J. Y. Fu, C. X. Jiang, M. Y. Wu, R. Y. Mei, A. F. Yang, H. P. Tao, X. J. Chen, J. Zhang, L. Huang, X. F. Zhao

**Affiliations:** ^1^ Institute of Developmental and Regenerative Biology, Zhejiang Key Laboratory of Organ Development and Regeneration, College of Life and Environmental Sciences, Hangzhou Normal University, Hangzhou, China; ^2^ Department of Physiology, Research Center of Neuroscience, College of Basic Medical Science, Chongqing Medical University, Chongqing, China; ^3^ Theabio Co., Ltd., Hangzhou, China; ^4^ Department of Horticulture, Zhejiang University, Hangzhou, China

**Keywords:** theabrownin, oligodendroglioma, astrocytoma, cell cycle, apoptosis

## Abstract

Theabrownin (TB), a natural compound present in the fresh leaves of green tea, is a potential antitumor agent. However, so far whether and how TB affects glioma is unclear. In this study, we investigated the effect of TB on astroglioma and oligodendroglioma cells. Surprisingly, TB significantly reduced the viabilities of HOG and U251 cells in a dose-dependent manner, which was accompanied by the upregulation of active-Casp-3, Bax, and PTEN; meanwhile, the antiapoptotic gene Bcl-2 was downregulated. In addition, TB treatment induced cell cycle arrest at the G1 and G2/M phases in HOG and U251 cells, respectively. TB treatment caused the downregulating of c-myc, cyclin D, CDK2, and CDK4 and upregulating of p21 and p27 in the HOG cell, while TB increased P53, p21, and p27 levels and decreased the levels of cell cycle regulator proteins such as CDK and cyclin A/B in the U251 cells. Therefore, the c-myc- and P53-related mechanisms were proposed for cell cycle arrest in these two glioma cell lines, respectively. Overall, our findings indicated that TB could be a novel candidate drug for the treatment of gliomas.

## Introduction

Gliomas are specialized tumors arising from glial cells in the central nervous system (CNS), which can be divided into astrogliomas, oligodendrogliomas, and mixed oligoastrocytomas (Dolecek et al., 2012). Generally, these gliomas are classified into different grades based on the growth rates: grade I tumors such as pilocytic astrocytomas and more common infiltrating gliomas; grade II oligodendrogliomas and astrocytomas; grade III anaplastic oligodendrogliomas, anaplastic astrocytomas, and anaplastic oligoastrocytomas; and grade IV glioblastomas (GBM). Glioma is one of the most challenging cancers to manage with a relative 5-year survival rate of about 5% (Ostrom et al., 2014). Despite the variety of modern therapies against GBM, it is still a deadly disease with extremely poor prognosis. Patients usually have a median survival of approximately 14 to 15 months after diagnosis (Thakkar et al., 2014; Lee 2016). The high fatality rates and low survival rates of glioma make it one of the deadliest and incurable cancers (Bray et al., 2018). Besides surgery, the widely used radiotherapy and chemotherapy have some limitations in cancer chemotherapy including the high cost of treatment (Penny and Wallace, 2015), multidrug resistance (Chi et al., 2017), and cytotoxicity to healthy tissues (Hersberger et al., 2013). Therefore, it is urgent to seek for more effective and low toxic agents for the glioma treatment.

Natural compounds (e.g., alkaloids, terpenoids, flavonoids, and polyphenols) are less toxic and exhibit diverse biological activities, which are turning popular in the field of drug discovery (Gullett et al., 2010; Millimouno et al., 2014). Green tea produced from the fresh leaves of *Camellia sinensis* (L.) O. Kuntze (Theaceae) is a traditional and commonly consumed nonalcoholic beverage in the world. Green tea has been reported to possess antioxidant, anti-inflammatory, antiproliferative, and antiangiogenic effects, which are beneficial for the prevention and treatment of various forms of diseases. To be noted, the anticancer effect of green tea has been evidenced by multiple studies (Imai et al., 1997; Yang et al., 2000; Cabrera et al., 2006; Suqanuma et al., 2011). Theabrownin (TB), theaflavin (TF), and thearubigin (TR) are three main tea pigments determining the color, taste, and the bioactivity of the tea liquor (Roberts et al., 1957). TB, as a main tea pigment, is a brown pigment with multiple aromatic rings and attached residues of polysaccharides and proteins (Roberts et al., 1957). The major functional groups in TB are carboxyl, hydroxyl, amino, and methyl. TB comprises a family of macromolecules transformed from polyphenols and is considered superior to TF or TR in physicochemical and medicinal properties. Previous studies demonstrated that TB possessed strong antiproliferative, proapoptotic, and cell cycle–arresting effects on human osteosarcoma and lung carcinoma (Wu et al., 2016; Zhou et al., 2017; Jin et al., 2018). In view of the TB’s key role in green tea, it can be expected that TB has a certain anticancer potential. However, the effect of TB on the glioma is lacking evidence.

In this study, we employed human glioma cell lines including astrocyte and oligodendrocyte cell-type glioma (A172, U87, U251, and HOG) to evaluate the effect of TB on these cell models and dissect the underling mechanisms. We found that TB significantly reduced the viabilities of HOG and U251 cells in a dose-dependent manner and induced cell cycle arrest at G1 and G2/M phases in HOG and U251 cells, respectively. Our results provide a promising candidate of natural products for glioma treatment.

## Materials and Methods

### Cell Line and Culture

Human glioma cell lines (A172, U87, U251, and HOG) were applied in this study (Table 1). All cell lines were cultured in DMEM containing 10% FBS at 37°C in a humidified 5% CO_2_ incubator. The medium was replaced daily, and the cells were treated with TB in different concentrations (0, 5, 25, 50, 100, 150, 200, and 300 μg/mL).

**TABLE 1:** Human glioma cell lines used in this study.

**Cell line**	**Cell type**	**Genus**	**Method used to generate cell lines**
**A172**	Astrocyte	Human	Clone derived from a surgically removed solid glioblastoma
**U87**	Astrocyte	Human	Clone derived from a surgically removed malignant astrocytoma
**U251**	Astrocyte	Human	Derived from a malignant glioblastoma tumor
**HOG**	Oligodendrocyte	Human	Clone derived from a surgically removed human oligodendroglioma

### Cell Viability Assay

The viability of glioma cells (A172, U87, U251, and HOG) upon exposure to TB was determined by CCK-8 assay, according to the provider’s instructions. In brief, cells were seeded in 96-well microplates and grown in the cell culture media (100 μL/well) at a density of 5 × 10^3^ cell/ml for 48 h. Afterward, the cells were incubated with TB solutions with different concentrations for 48 and 72 h, respectively. The samples were washed once with the incomplete cell culture media to remove the residual TB, followed by adding 10 μL of CCK-8 reagents and 100 μL of incomplete cell culture media to each well to allow the cells to grow for another 1 h in a 5% CO_2_-humidified atmosphere at 37°C. Finally, the microplates were analyzed with a microplate reader, and the absorbance value at 450 nm (OD_450_) for each well was measured. The viability of cells was expressed as the percentage of the cell survival rate between the experimental group and the control group. The value for each treatment represents the averaged value taken from three replicate wells in three independent experiments.

### Cell Morphology and DAPI Staining

The TB-treated HOG and U251 cells at 24 h were washed with phosphate-buffered saline (PBS) thrice and fixed with 4% paraformaldehyde in PBS for 30 min at room temperature. Then, the cells were permeabilized with 0.5% Triton X-100 in PBS for 10 min. An aliquot of the cells was mounted using a ProLong® Diamond Antifade Mountant with DAPI in the dark. The stained cells were observed under a fluorescence microscope. Three coverslips were used as replicates of each group, and the apoptotic nuclei of the cells were visualized.

### Cell Cycle Analysis

Cell cycle analysis was performed with flow cytometry. In brief, U251 and HOG cells were cultured on six-well plates with a density of 3 × 10^5^ cells/well for 24 h, followed by TB treatment for 72 h. The cells were harvested and washed with PBS thrice and suspended in cold phosphate-buffered saline. Cells were then stained with PI/RNase staining solution (20 μg/ml PI and 10 μg/ml DNase-free RNase) at 37°C in the dark for 30 min. The cell cycle was analyzed in triplicate using flow cytometry.

### Real-Time PCR Analysis

After TB treatment, the gene expression in HOG and U251 cells was detected by using real-time PCR assay on an ABI QuantStudio^TM^ 7 Flex Real-Time PCR System (Applied Biosystems, Foster City, CA, United States). The total RNA of the cells in each group was extracted using Trizol reagent and converted to cDNA via reverse transcription. At the end of each reaction, a melting curve analysis was performed. β-actin was used as the reference gene, and the 2^−ΔΔCT^ method was applied to analyze the relative expression of each gene (Table 2).

**TABLE 2:** Primer sequences used for qPCR.

**Gene**	**Forward primer**	**Reverse primer**
Caspase-3	GAC​TGC​GGT​ATT​GAG​ACA​GA	CGA​GTG​AGG​ATG​TGC​ATG​AA
Bcl-2	AAC​ATC​GCT​CTG​TGG​ATG​AC	GAG​CAG​CGT​CTT​CAG​AGA​CA
Bax	GAA​TTG​GCG​ATG​AAC​TGG​AC	GCA​AAG​TAG​AAA​AGG​GCA​ACC
P27	TGG​AAA​GCG​GTC​TGC​AAG​TG	TCA​CTG​TCA​CAT​TCA​GGG​GC
P21	GGA​TGT​CCG​TCA​GAA​CCC​AT	CCC​TCC​AGT​GGT​GTC​TCG​GTG
PTEN	CCG​AAA​GGT​TTT​GCT​ACC​ATT​CT	AAA​ATT​ATT​TCC​TTT​CTG​AGC​ATT​CC

### Western Blot Analysis

Following TB treatment, HOG and U251 cells were harvested and washed twice with cold PBS. Then, the cells were lysed with radioimmunoprecipitation assay (RIPA) buffer (P0013B, Beyotime, CN) containing protease inhibitor (C600386, Sangon Biotech, CN) and phosphatase inhibitor (C500017, Sangon Biotech, CN) for 30 min on ice, with repeated freezing and thawing for three times. The lysates were then sonicated and centrifuged at 12,000 rpm for 15 min at 4°C to obtain the total protein supernatant. Protein concentrations were determined by a commercial BCA kit (P0010, Beyotime, CN). Appropriately 20 μg of lysed protein was boiled in sample-loading buffer and separated by a denaturing 10% SDS-PAGE. The gels were transferred onto polyvinylidene fluoride membranes (Millipore, Billerica, MA, United States) and blocked with 5% nonfat milk for 1.5 h, followed by overnight incubation at 4°C with the following primary antibodies: c-Myc, cyclin A1, cyclin B1, cyclin D1, P53, p-P53, Cdk1, Cdk2, Cdk4, AKT, p-AKT, Erk, p-Erk, active caspase-3, Bax, Bcl-2, PTEN, and GAPDH. Following the incubation with peroxidase-conjugated goat anti-rabbit IgG or anti-mouse IgG at room temperature for 1 h, the membrane was visualized using BeyoECL Star (BEYOTIME, China), and pictures were taken using a ChemiDoc imaging system (BIO-RAD, United States).

### Measurement of Apoptosis

The apoptotic assay was performed using an annexin V-FITC apoptosis detection kit according to the manufacturer’s protocol (Vazyme Biotech Co., Ltd., Nanjing, China). For the flow cytometric analysis, cells were harvested with 0.05% trypsin with EDTA, washed twice with cold PBS, and resuspended in 500 μL binding buffer supplied by the manufacturer. The cells were then incubated with 5 μL annexin V-FITC (40 μg/mL) and 5 μL propidium iodide (PI) (40 μg/mL) in the dark for 10 min. Analysis was performed using a BD FACSAria^TM^ flow cytometer (Becton Dickinson, San Jose, CA, United States) set at an excitation wavelength of 488 nm and an emission wavelength of 605 nm.

### TUNEL Assay

Cells were planted into 48-well plates. After 24 h, cells were treated with TB (0, 25, and 50 μg/mL) at 37°C in a humidified 5% CO_2_ for 24 h. Apoptosis was evaluated using a TUNEL FITC Apoptosis Detection Kit (Vazyme, Nanjing, CN) according to the manufacturer’s protocol. In brief, cells were incubated with 20 μg/ml proteinase K for 20 min at room temperature and washed in PBS. The sections were then incubated with terminal deoxynucleotidyl transferase and FITC-12-dUTP at 37°C for 1 h. Finally, the sections were washed in PBS three times and counterstained with DAPI. Images of TUNEL staining were acquired with a fluorescence microscope.

### Statistical Analysis

For each analysis, results from independent TB were treated as biological replicates (n ≥ 3). Quantitative data were presented as mean ± SD. Statistical significance was evaluated by Student’s t-test. Differences were considered statistically significant at *p < 0.05, **p < 0.01, and ***p < 0.001 (n.s.: no significant difference).

## Results

### TB Inhibits Cell Viability of Glioma Cells

**FIGURE 1:**
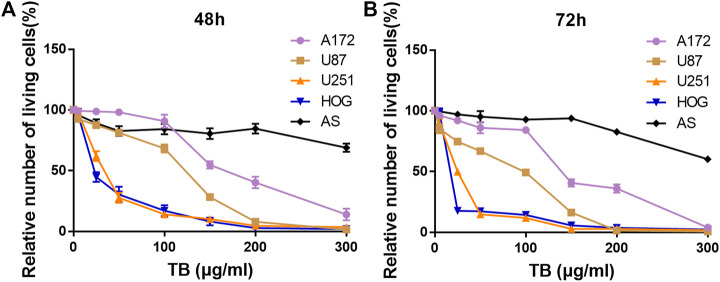
Effect of TB on cell viability of four glioma cell lines and the control primary culture astrocyte for 48 and 72 h determined by CCK-8 assay. **(A)** Effect of TB on cell viability of four glioma cell lines and the control primary culture astrocyte for 48 h determined by CCK-8 assay. **(B)** Effect of TB on cell viability of four glioma cell lines and the control primary culture astrocyte for 72 h determined by CCK-8 assay. Values are presented as mean ± SD (*n* = 3).

We treated four human glioma cell lines and primary cultured astrocyte with various concentrations (0–300 μg/ml) of TB and conducted cell viability by using CCK-8 assay at 48 and 72 h (Figure 1). We found that TB treatment caused an obvious inhibition of the cell survival rate of four glioma cells at various concentrations. The inhibitory effect was at a dose- and time-dependent manner of TB treatment. After TB treatment for 48 h, the IC_50_ values declined to 268, 124, 36, and 26 μg/ml for A172, U87, U251, and HOG cells, respectively (Figure 1A), and the IC_50_ values declined to 142, 98, 27, and 17 μg/ml for A172, U87, U251, and HOG cells, respectively, after TB treatment for 72 h (Figure 1B). However, there were no significant changes in the primary cultured astrocyte, which was regarded as a control group (Figure 1). The effect of TB on U251 and HOG cell viability inhibition was more efficacious than others; therefore, these cell lines were selected for the following assay; 75 μg/ml was selected as a high effective dose of TB for the following assays.

### TB Induces Glioma Apoptosis

**FIGURE 2:**
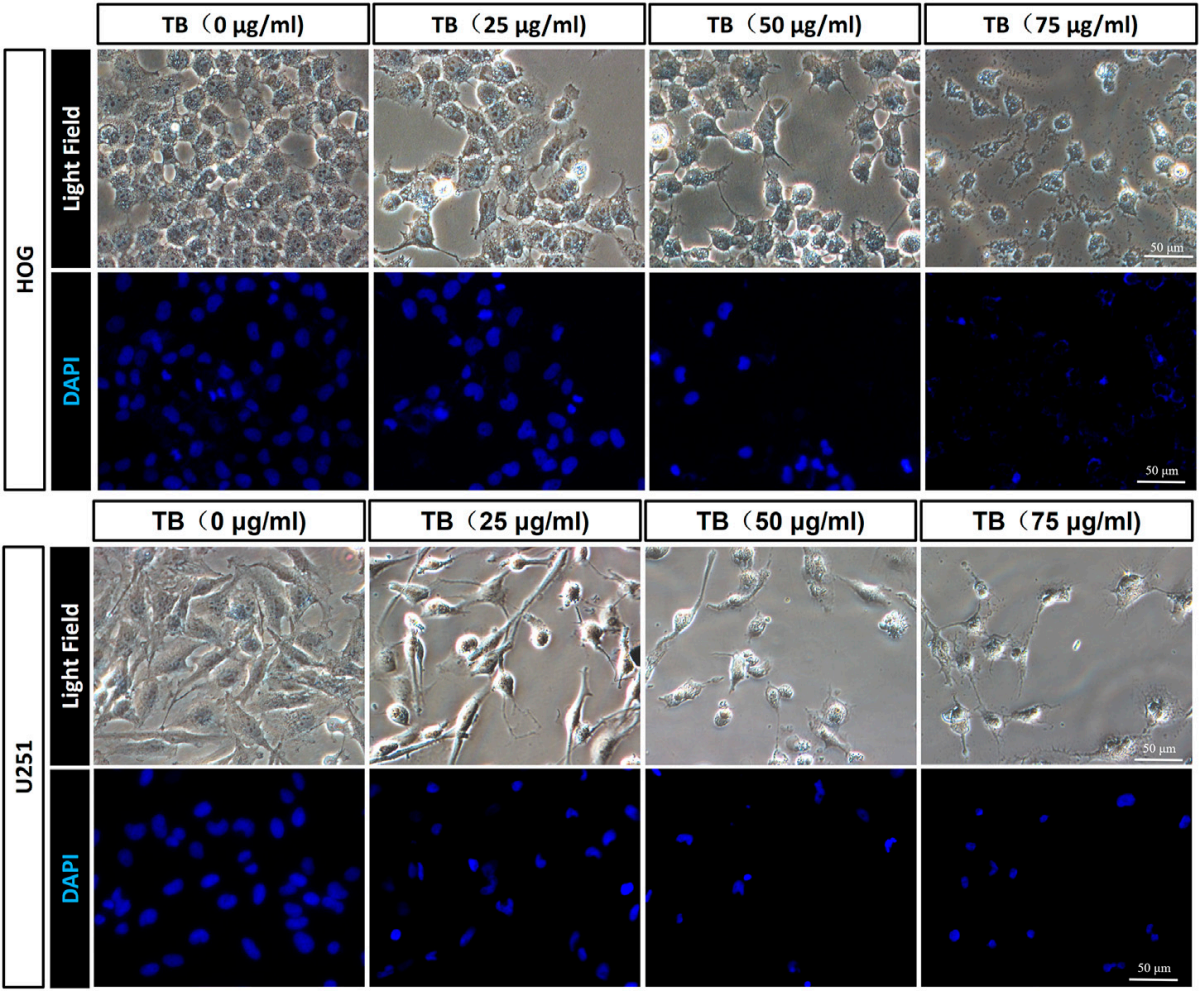
Morphological observation on TB-treated HOG and U251 cells by DAPI staining. Scale bar: 50 μm.

We performed fluorescence staining for apoptotic morphology observed after 48 h TB treatment. Compared to the control group, TB treatment significantly increased cell detachment both in HOG and U251 cells. The TB treated cells showed shrunken shape, chromatin condensation, karyopyknosis, and nuclear fragmentation in the nucleus, which are typical features of apoptotic cells (Figure 2).

**FIGURE 3:**
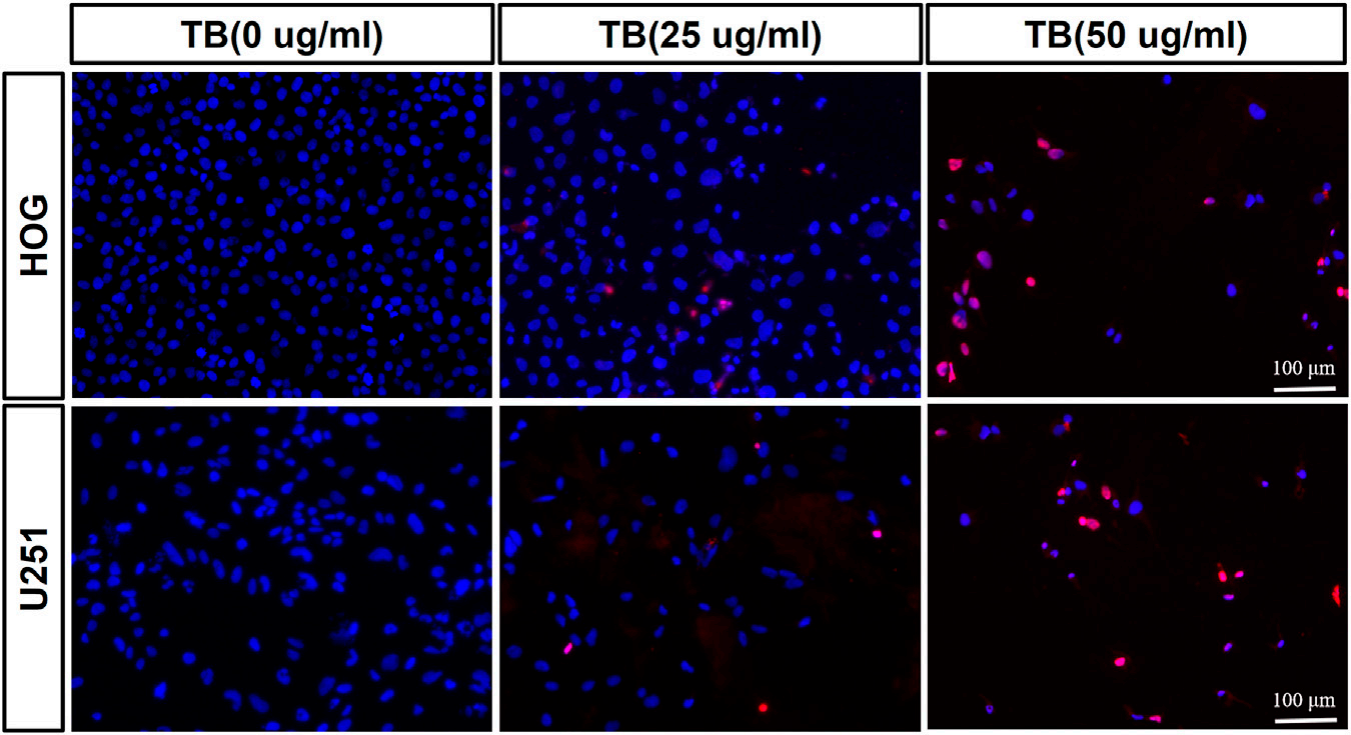
Observation of TB-induced apoptosis on HOG and U251 cells by TUNEL assay. Scale bar: 100 μm.

**FIGURE 4:**
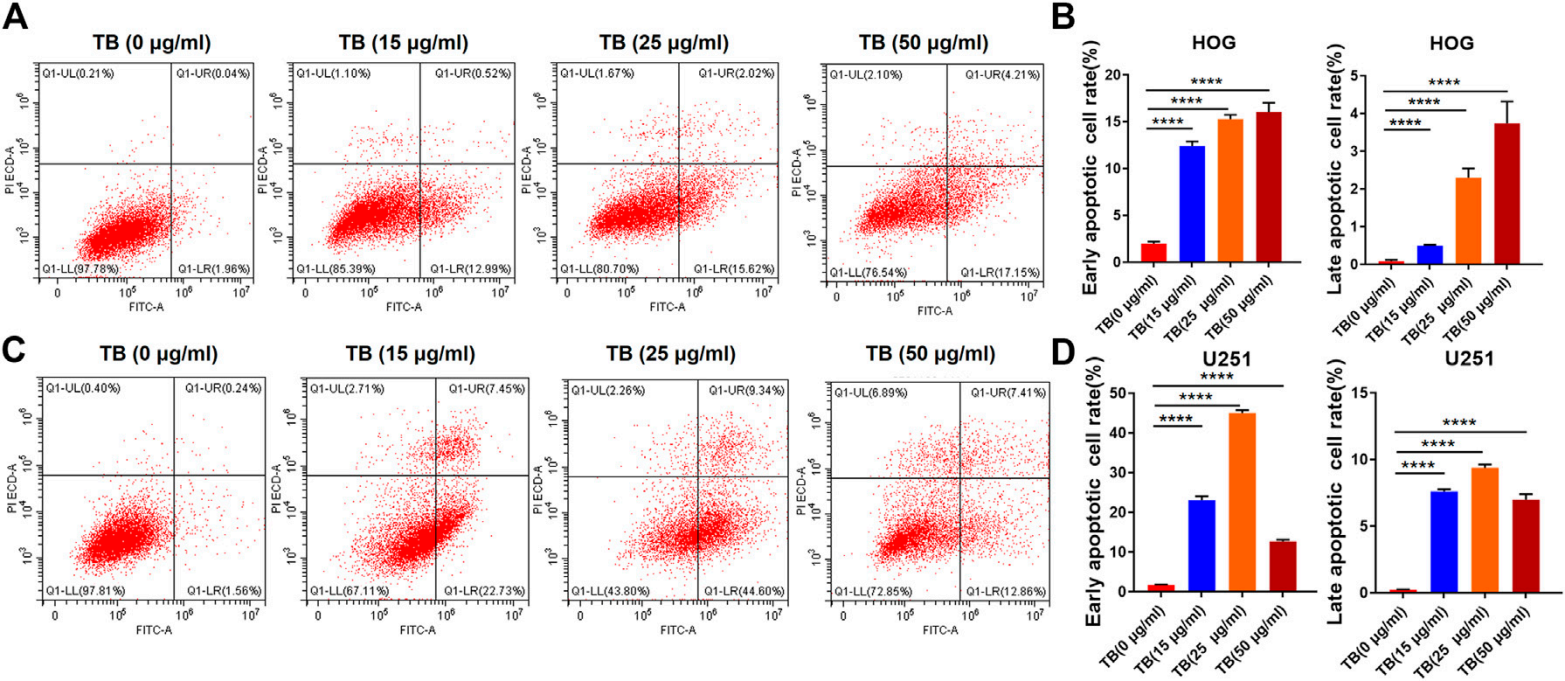
**(A)**, **(B)**: Flow cytometry analysis of HOG and U251 cells apoptosis by double staining with Annexin V-FITC and propidium iodide (PI). **(C)**: Quantitative analysis of apoptosis induced by TB in HOG cells. **(D)**: Quantitative analysis of apoptosis induced by TB in U251 cells.

Transferase dUTP nick-end labeling assay was performed to evaluate TB-induced apoptosis of HOG and U251 cells. As shown in Figure 3, there was no positive signal in the control group cells, whereas increased apoptosis signal was observed in the TB treated cells and with strong fluorescence staining in a TB concentration-dependent manner. Flow cytometry using Annexin V-FITC/PI double staining was further performed to validate the apoptosis-inducing effect of TB on glioma cells. As shown in Figure 4, a significant increase of early apoptosis and a progressive increase of late apoptosis were found with increasing concentration of TB treatment on HOG and U251 cells at 48 h (Figures 4A,C). The percentages of apoptotic cells increased from 12.41 ± 0.48% to 16.04 ± 0.98% (early) 0.50 ± 0.02% to 3.73 ± 0.58% (late), following treatment with 15, 25, and 50 μg/ml TB on HOG, respectively (Figure 4B). And the percentages of apoptotic cells increased from 23.07 ± 0.1% to 45.01 ± 0.73% (early) 7.60 ± 0.15% to 9.38 ± 0.25% (late), following treatment with 15, 25, and 50 μg/ml TB on U251, respectively (Figure 4D). Consistently, quantitative analysis showed that as compared with the control group, the percentage of apoptotic cells in the TB-treated group was significantly increased in a dose-dependent manner.

**FIGURE 5:**
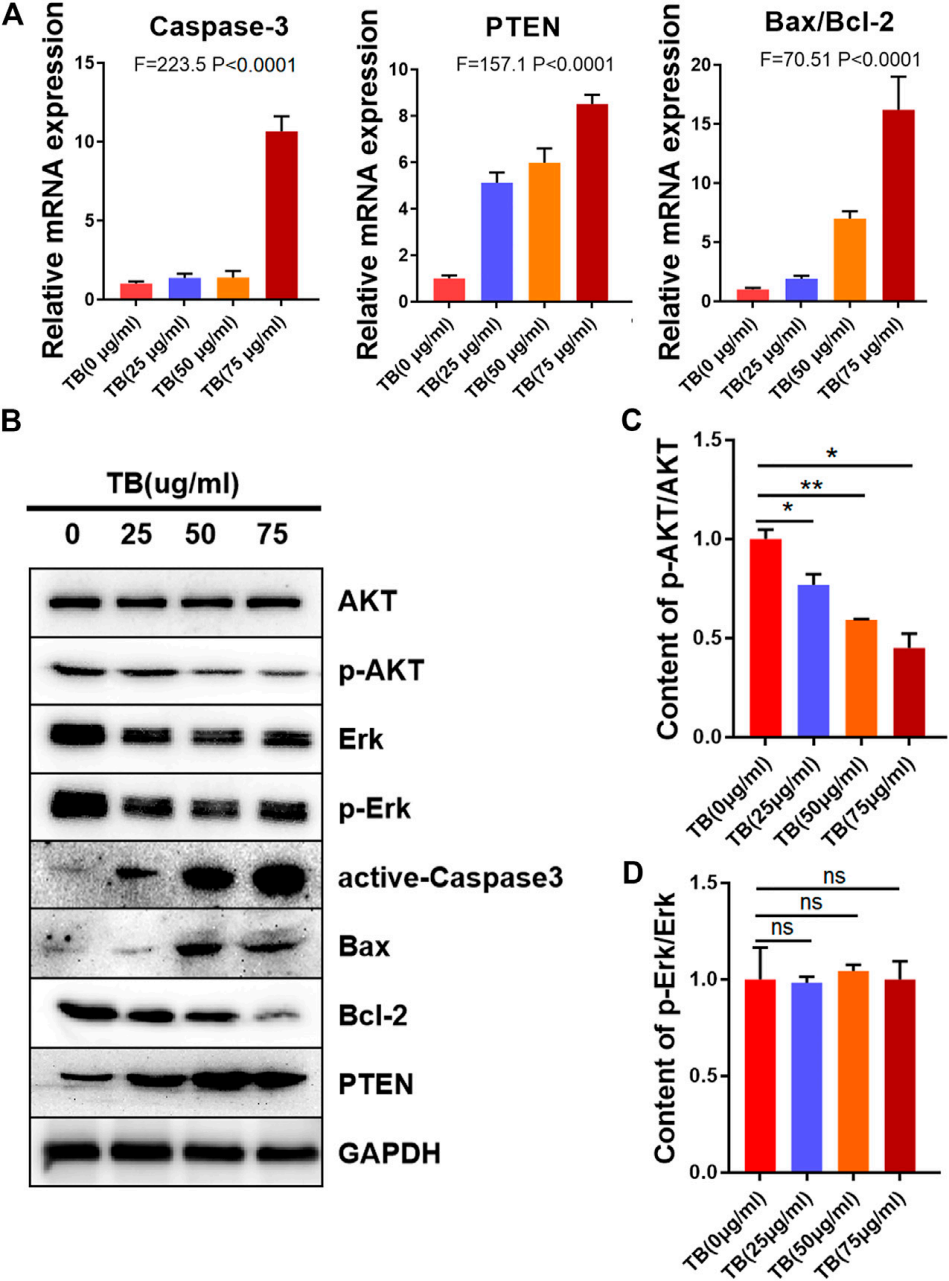
Relative mRNA and protein expression of target genes in HOG cells with 48-h TB treatment at 25, 50, and 75 μg/ml. Values are presented as mean ± SD of three replicates. **(A)**: Relative mRNA expression of target genes in HOG cells with 48 h TB treatment at 25, 50, and 75 μg/ml. **(B)**: Relative protein expression of target genes in HOG cells with 48 h TB treatment at 25, 50, and 75 μg/ml. Values are presented as mean ± SD of three replicates. **(C)**: Quantitative analysis the protein expression of p-AKT/AKT in HOG cells. **(D)**: Quantitative analysis the protein expression of p-ERK/ERK in HOG cells.

**FIGURE 6:**
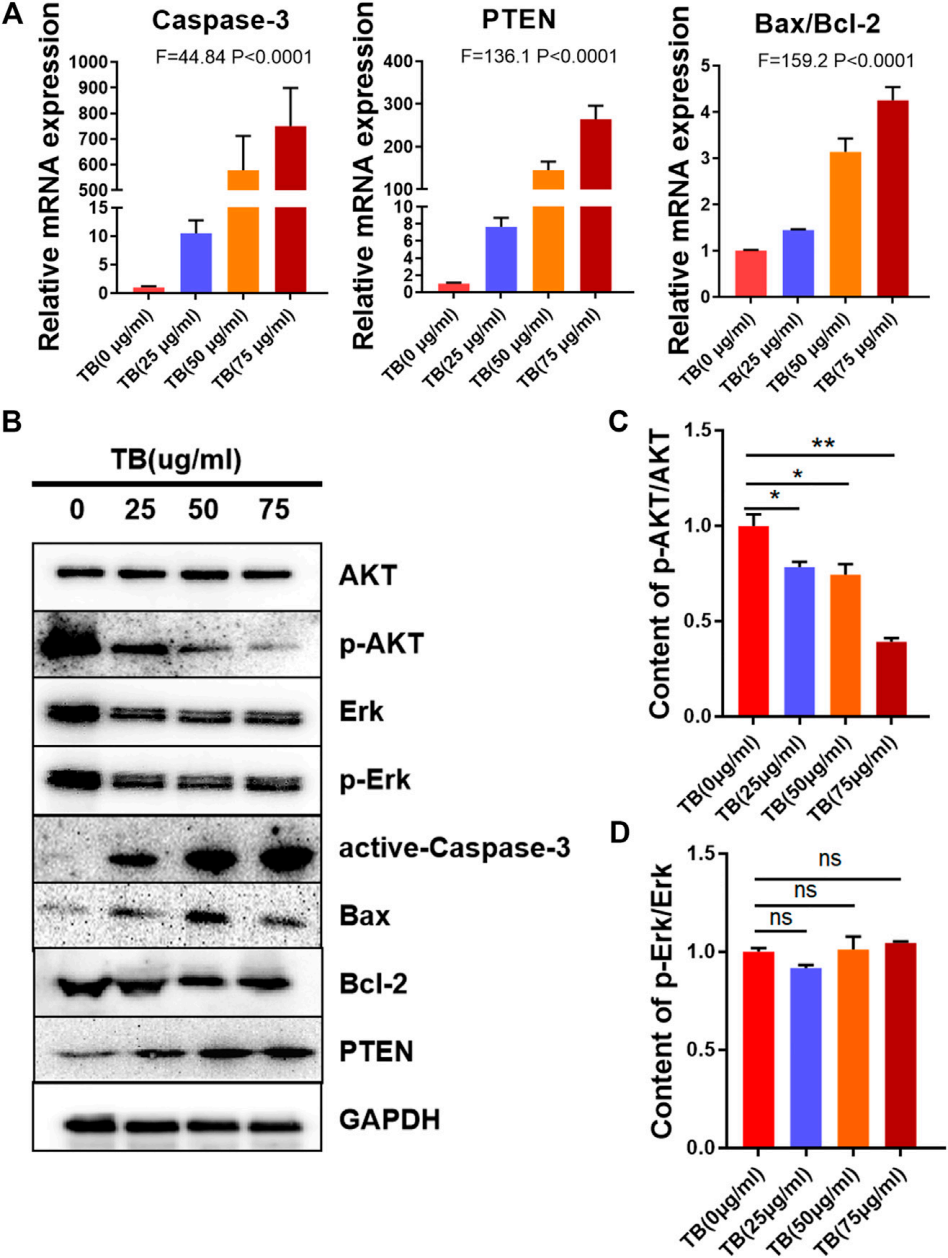
Relative mRNA and protein expression of target genes in U251 cells with 48-h TB treatment at 25, 50, and 75 μg/ml. Values are presented as mean ± SD of three replicates. **(A)**: Relative mRNA expression of target genes in U251 cells with 48 h TB treatment at 25, 50, and 75 μg/ml. **(B)**: Relative protein expression of target genes in U251 cells with 48 h TB treatment at 25, 50, and 75 μg/ml. Values are presented as mean ± SD of three replicates. **(C)**: Quantitative analysis the protein expression of p-AKT/AKT in U251 cells. **(D)**: Quantitative analysis the protein expression of p-ERK/ERK in U251 cells.

To determine the mechanisms underlying the TB-induced glioma cell apoptosis, several important apoptosis-related genes and proteins were analyzed by qPCR and Western blot. As shown in Figures 5, 6, TB treatment caused acute upregulation of active caspase-3, Bax, and PTEN mRNA expression in a dose-dependent manner both in the HOG and U251 cells, as well as an increase of the proapoptotic gene Bax and downregulated the antiapoptotic gene Bcl2 (Figures 5A, 6A). The Western blot analysis showed similar results that TB treatment upregulated the protein expression of active caspase-3, Bax, and PTEN; meanwhile, the protein expression of antiapoptotic gene Bcl-2 was downregulated (Figures 5B, 6B). Interestingly, TB treatment downregulated the phosphorylation level of AKT in both cell lines but did not affect the phosphorylation level of Erk (Figures 5C,D, 6C,D).

### TB Induces Cell Cycle Arrest in Glioma

**FIGURE 7:**
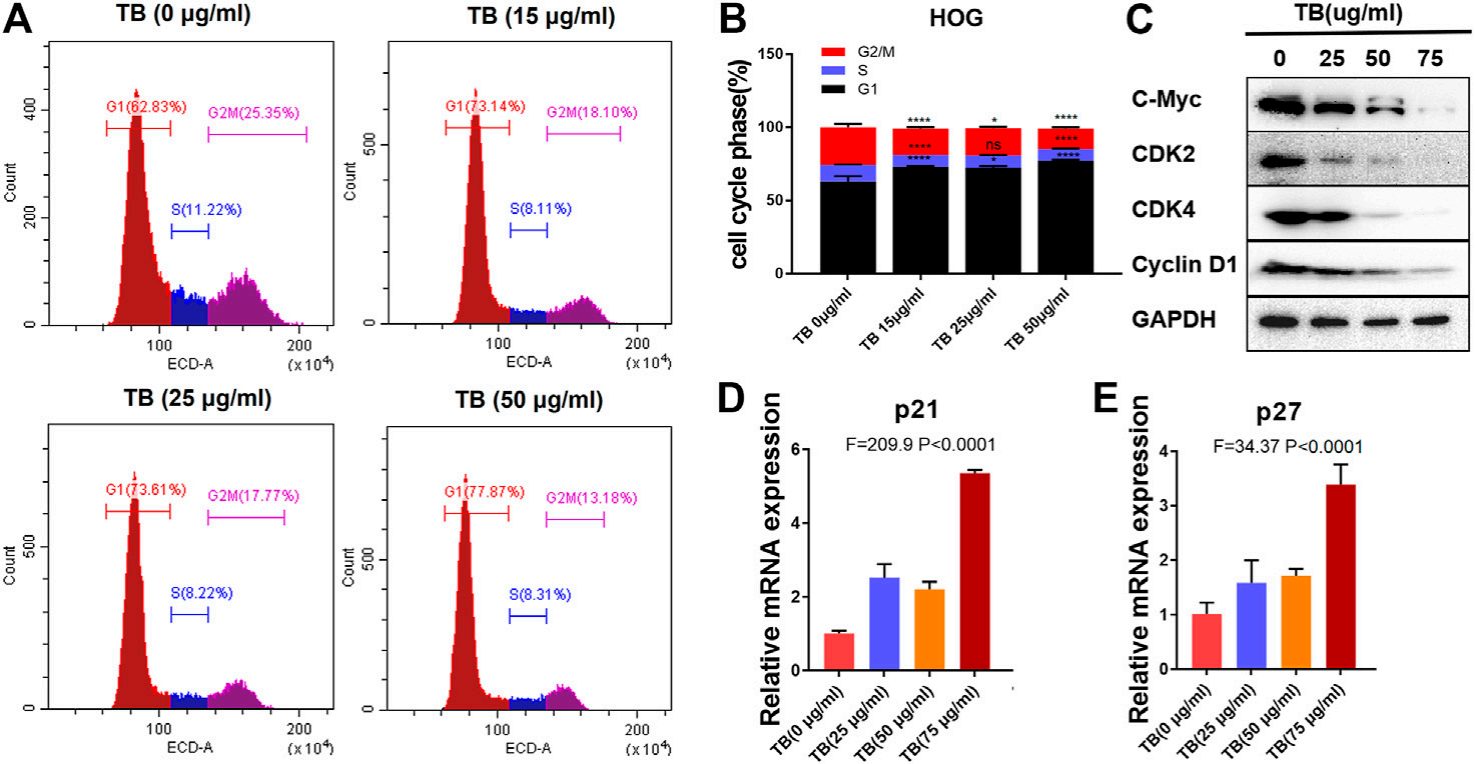
**(A)**: Effect of TB on the cell cycle of HOG cells determined by flow cytometry. **(B)**: Quantitative analysis of cell cycle arrest induced by TB in HOG cells. **(C)**: Relative protein expression of target genes in HOG cells with 48 h TB treatment at 25, 50, and 75 μg/ml. **(D, E)**: Relative mRNA expression of p21 and p27 in HOG cells with 48 h TB treatment at 25, 50, and 75 μg/ml. Values are presented as mean ± SD of triplicate.

**FIGURE 8:**
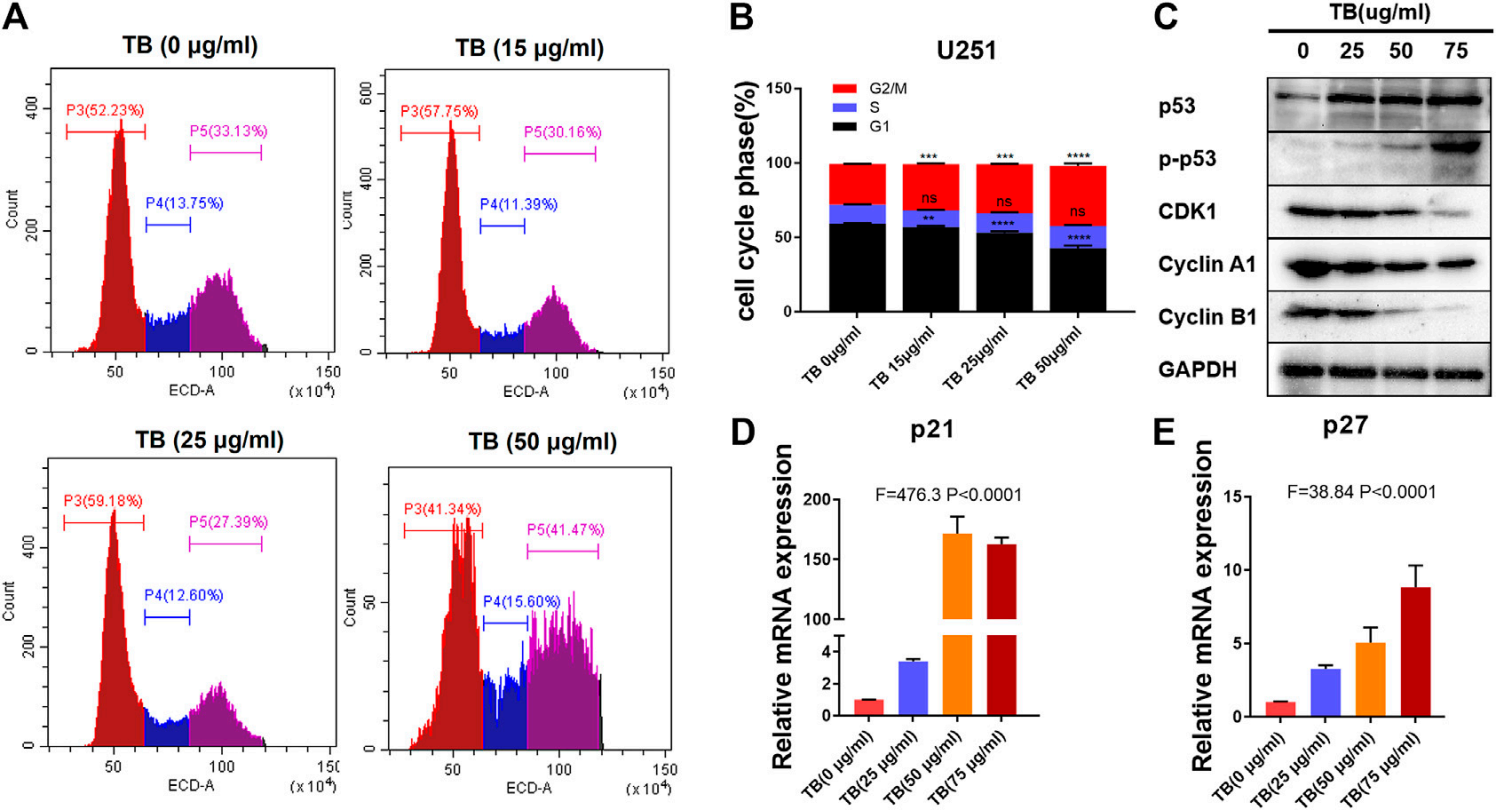
**(A)**: Effect of TB on the cell cycle of U251 cells determined by flow cytometry. **(B)**: Quantitative analysis of cell cycle arrest induced by TB in U251 cells. **(C)**: Relative protein expression of target genes in U251 cells with 48 h TB treatment at 25, 50, and 75 μg/ml. **(D, E)**: Relative mRNA expression of p21 and p27 in U251 cells with 48 h TB treatment at 25, 50, and 75 μg/ml. Values are presented as mean ± SD of three replicates.

To further confirm the effects of TB on the cell cycle in HOG and U251 cells, these two cell lines were treated with various concentrations of TB and then were stained with PI for cell cycle analysis. As shown in Figure 7, TB-treated HOG cells were accumulated in the G1 phase of the cell cycle, with a significant increase of the cell number in the G1 phase as compared to no TB-treated controls (Figure 7A). The number of cells in the S phase and G2/M phase significantly decreased, except for that in the G1 phase at 72 h. Compared with the control group, the percentage of number of cells in the G1 phase increased from 62.85 ± 3.65% to 77.22 ± 0.57% at a concentration of 50 μg/m, while that in the G2/M phase decreased (Figure 7B). The expression of CDK2, CDK4, c-myc, and cyclin D1 was decreased at the protein level with increased TB concentration in HOG cells (Figure 7C). Meanwhile, the cyclin-dependent kinase inhibitor p21/27, which suppresses the phosphorylation of CDK/2/4, was upregulated after TB treatment (Figure 7D,E), implying that p21/27 was involved in the G1 arrest induced by TB. Interestingly, TB-treated U251 cells were accumulated in the G2/M phase of the cell cycle, with a significant increase of the cell number in the G2/M phase as compared to controls (Figure 8A). The results showed that the number of cells in the S phase and G1 phase significantly decreased at 72 h, but the number of cells in the G2/M phase increased. And the percentage of the number of cells in the G2/M phase increased from 27.22 ± 0.25% to 40.18 ± 1.83% at a concentration of 50 μg/m, while that in the G1 phase decreased (Figure 8B). Consistently, Western blot results showed that CDK1, cyclinA1, and cyclin B1 protein levels were reduced, while p53 and the phosphorylated P53 (Ser20) were increased with a dose-dependent manner (Figure 8C). In addition, the cyclin-dependent kinase inhibitor p21/27 was also upregulated (Figure 8D,E). These trends indicated that TB could induce cell cycle arrest of glioma cells in the gap phase and was cell line–specific.

## Discussion

Because of significant disadvantages of cancer chemotherapy including the high cost of treatment (Penny and Wallace, 2015), multidrug resistance (Chi et al., 2017), and cytotoxicity to healthy cells (Hersberger et al., 2013), there has been a growing interest in using natural compounds as a single agent or combination therapy for anticancer treatment, owing to their advantages of high bioactivities and low toxicity. Green tea is one of the plant sources that are rich in natural compounds such as polyphenols. And TB is mainly composed of polyphenols that have been found with anticancer activity against various cancers (Khan et al., 2009; Siddiqui et al., 2009; Yang et al., 2009; Wu et al., 2016).

Here, we demonstrated that TB significantly inhibited not only astrocyte but also oligodendrocyte cell-type glioma cell viability (Figure 1) in a dose-dependent manner and blocked them in different cell cycles at the G0/G1 phase and G2/M phase, respectively (Figures 7, 8). Morphological observation, DAPI staining, and TUNEL assay revealed typical cell apoptotic characteristics in TB-treated glioma cells (Figures 2, 3), and annexin-V/PI flow cytometric analysis further evidenced the apoptosis-inducing effect of TB (Figure 4). In addition, the real-time PCR and Western blot analyses showed that TB induced cell apoptosis through upregulation of active Casp-3, Bax, and PTEN, while Bcl-2 through downregulation (Figures 5, 6). Caspases (cysteine–aspartic acid proteases) are key mediators of apoptosis, and most cell apoptosis-inducing factors eventually cause cell apoptosis through the caspase-mediated signal transduction pathway (Xie and Yang, 2014). Proapoptotic and antiapoptotic members such as Bax and Bcl-2, respectively, are important for regulation of cell death and apoptosis that respond to anticancer therapy (Youle and Strasser, 2008). The ratio of proapoptotic and antiapoptotic members determines cell fate, survival, or death, when exposed to apoptotic stimuli (Oltvai et al., 1993).

Furthermore, we also found the upregulation of PTEN in the glioma cells after TB treatment. PTEN encodes a lipid phosphatase that antagonizes the phosphatidylinositol-3-kinase (PI-3K) signaling pathway by converting phosphatidylinositol 3, 4, 5-trisphosphate (PIP3) into phosphatidylinositol 4, 5-bisphosphate (PIP2). PTEN negatively regulates the PI-3K pathway by converting PIP3 back into PIP2. PIP3 functions as a second messenger by recruiting Akt and phosphoinositide-dependent protein kinase (PDK)-1 to the plasma membrane, resulting in phosphorylation of Akt on Thr308. Phosphorylated Akt activates downstream targets that regulate cell survival, proliferation, and cell growth. In humans, PTEN is a crucial tumor suppressor in many cancer types, and PTEN deletion is characteristic of many high-grade glial tumors (Zhang et al., 2010).

We further demonstrated that TB inhibited glioma cell viability in a dose-dependent manner and blocked the HOG cell cycle at the G0/G1 phase and the U571 cell cycle at the G2/M phase (Figures 7, 8). The real-time PCR and Western blot analyses have clarified the associated mechanism that TB induced cell cycle arrest through downregulation of c-Myc, cyclin D, CDK2, and CDK4 and upregulation of p21 and p27 in the HOG cell (Figure 7). The c-myc is a transcriptional factor and a well-known oncogene expressed in the G1 phase of the cell cycle; downregulated c-myc can decrease cell proliferation and result in cell cycle arrest (Nesbit et al., 1999; Delmore et al., 2011). It is well-known that cyclin D, p21, and p27 are downstream genes targeted by c-Myc for cell cycle regulation. The cyclin D is a member of the cyclin family and the major G1 phase cyclin, and the complex of cyclin D and cdk4 is necessary to regulate G1/S transition in the cell cycle progression (Sherr, 1996). Our results showed both declined cyclin D and cdk4, suggesting that G1/S transition cell cycle progression was retarded in the TB-treated HOG cells. Furthermore, the CDK inhibitors P21 and P27, capable of binding to and inhibit the active cyclin/CDK complexes in the nucleus to induce blockade of G1/S transitions of the cancer cell cycle (Grandori et al., 2000; Kuilman et al., 2010), were increased in the TB-treated HOG cells. Therefore, a mechanism can be proposed that TB directly downregulated the c-Myc and then transcriptionally regulated the downstream genes through the CDK inhibitors P21 and P27 to induce the oligodendroglioma cell HOG cell cycle arrest.

On the other hand, we also demonstrated that TB caused an accumulation of astrocyte cell-type glioma cells U251 in the G2/M phase. As we know, p53 is an important tumor suppressor gene and a major negative regulator in cell growth and cell cycle progression. The phosphorylation of p53 in Ser20 activates p53 as a transcription factor and mediates its cellular stabilization in response to DNA damage (Chehab et al., 1999; Nakamura, 2009). Here, we found that TB treatment increased P53 and P21/27 levels and decreased the levels of the cell cycle regulator proteins CDK and cyclin A/B. It had demonstrated that activation of p53 can upregulate p21/27 expression, which promotes G2/M cell cycle arrest (Lloyd et al., 1997; Boulaire et al., 2000; Allan and Clarke, 2007). These results suggested that TB induced arrest of the G2/M phase by CDK1 via a p53-dependent p21/27 upregulation. It is believed that cell cycle arrest in the G1 phase is of potentially great clinical value because the G1 phase is the most sensitive time point for cancer therapy. Since the cell cycle restriction point occurs in the mid-G1 phase, cancer cells become independent of growth factors and become committed to cell division after this point (Kastan and Bartek, 2004).

**FIGURE 9:**
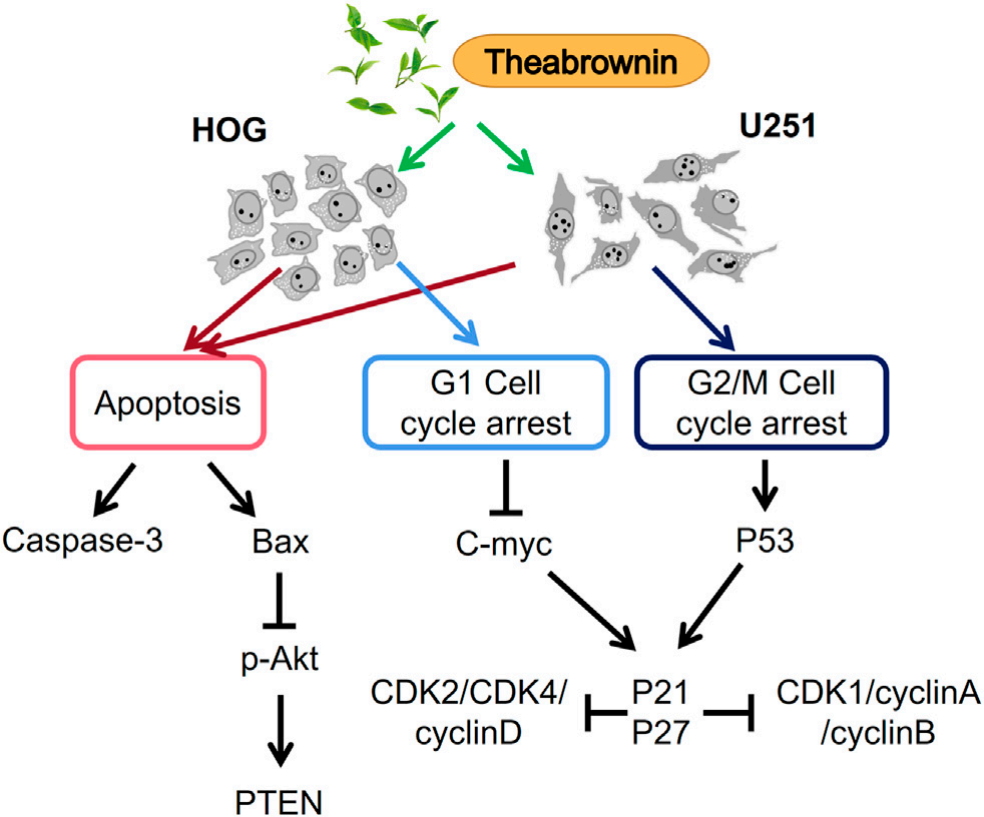
Apoptosis-inducing and cell cycle arrest mechanism of TB on HOG and U251 cells.

Overall, in this study, we found TB inhibited cell proliferation and induced apoptosis of glioma cells through active caspase3 and AKT mechanisms (Figure 9). TB induced cell cycle arrest by regulation of c-Myc or p53 and affects downstream genes through the CDK inhibitors P21 and P27 for HOG or U251, respectively (Figure 9). Our findings indicated TB acts as a new anticancer agent for the treatment of glioma, and further studies are warranted to confirm TB’s *in vivo* effect on the glioma and the underlying molecular mechanisms.

## Data Availability

The raw data supporting the conclusions of this article will be made available by the authors, without undue reservation.
